# Hospitalization for computer-assisted hexapod ring fixation application – analyses of patient variability, peri-operative complications, hospital costs, and discharge status

**DOI:** 10.1186/s12891-022-05171-6

**Published:** 2022-03-05

**Authors:** J. Spence Reid, Mollie Vanderkarr, Bidusee Ray, Abhishek Chitnis, Chantal E. Holy, Charisse Sparks

**Affiliations:** 1 Department of Orthopaedic Surgery, Penn State University College of Medicine, 500 University Drive, Hershey, PA 17033 USA; 2 DePuy Synthes, West Chester, PA USA; 3 Mu Sigma, Bengalore, India; 4 Real World Analytics and Research, Johnson & Johnson, NJ New Brunswick, USA

**Keywords:** Long bone deformities, Hexapod ring fixation, Hospital, Complications, Resource utilization, Costs

## Abstract

**Background:**

Computer-assisted hexapod ring fixation systems (HRF) are used for multiple conditions and in very diverse patient populations. This study analyzes perioperative outcomes following HRF application based on patient etiology and clinical presentation.

**Methods:**

Data from patients in the Premier Hospital Billing Database between 2007–2019 undergoing HRF application were analyzed for the duration of patients’ hospitalizations. Patients were grouped based on etiology: acquired deformity, arthrosis, congenital deformity, deep infection, infected nonunion, fracture, nonunion, and other post-operative complications. Demographics, comorbidities, operating room time (ORT), length of stay (LOS), peri-operative complications, and hospital costs were estimated using generalized linear models. Logistic regression evaluated factors associated with peri-operative complications.

**Results:**

One thousand eight hundred eighteen patients (average age: 46.9, standard deviation (SD) (19.6) – 38.9% female) were included in the study, and included 72% fracture cases, 9.6% deep infection, 10.2% deformity (acquired: 5.9%, congenital: 4.3%), 4.2% nonunions, 2% arthrosis and 1.4% other sequelas from prior fractures. Comorbidities varied across diagnosis categories and age, 40% adults and 86% pediatric had no comorbidities. Pediatric cases mostly suffered from obesity (16.1%) and pulmonary disease (10.7%). Complicated diabetes was present in 45.9% of arthropathy and 34.3% of deep infection patients. ORT, LOS and inflation-adjusted hospital costs for all patients averaged 277.7 min (95% Confidence interval (CI): 265.1–290.3), 7.07 days (95% CI: 6.6–7.5) and $41,507 (95%CI: $39,728-$43,285), respectively, but were highest in patients with deep infection (ORT: 369 min (95%CI: $321.0-$433.8); LOS: 14.4 days (95%CI: $13.7-$15.1); Cost: $54,666 (95%CI: $47,960-$63,553)). The probability of having an intraoperative complication averaged 35% (95%CI: 28%-43%) in adult patients with deep infection vs 7% (95%CI: 2%-20%) in pediatric cases treated for congenital deformity. The risk for intraoperative complications was mostly associated with preexisting comorbidities, an Elixhauser > 5 was the most predictive risk factor for complications (odds ratios: 4.53 (95%CI: 1.71–12.00, *p* = 0.002).

**Conclusions:**

There is important heterogeneity among HRF patients. Adults with HRF for fracture, deep infection and arthrosis are at far greater risk for peri-operative complications vs. patients with deformity, especially pediatric deformity cases, mostly due to existing comorbidities and age. Device-specific HRF clinical studies cannot be generalized beyond their exact patient population.

**Supplementary Information:**

The online version contains supplementary material available at 10.1186/s12891-022-05171-6.

## Background

The surgical indications for computer-assisted hexapod ring fixation systems (HRF) are broad but can be grouped into: acquired and congenital deformities, treatment of complex fractures, and post-traumatic complications such as nonunion with or without superimposed infection [1–9]. These conditions, if left untreated, can lead to secondary joint arthrosis, loss of range of motion, joint instability, pain and amputation [4–6, 10, 11]. HRF systems play an important role in the treatment of these complex etiologies as they allow gradual correction of severe deformity in patients with debilitating injuries or conditions, modification of treatment during correction, and minimization of neurovascular damage [12]. In addition, insufficient bone length from any diagnosis can be restored using the techniques of distraction osteogenesis as a part of the overall treatment plan. Compared to internal fixation, they also cause less disruption of the soft tissues, osseus blood supply, and periosteum [13]. Inherent in their designs, hexapod ring fixator systems require software to solve the complex mathematics necessary to precisely move one ring with respect to another. This increased accuracy of movement may potentially reduce the time required for correction and overall time in frame compared to more conventional methods not employing an HRF [14].

HRF systems consist of ring fixators and struts that can be manipulated in six degrees of freedom to gradually correct deformity and/or lengthen bones in patients with the conditions outlined above, using computer-assisted planning software [10, 15, 16]. There are at least six HRF systems currently available in the U.S. While generally successful, the treatment is demanding for both the surgeon and the patient. The surgeon must construct a stable frame and correctly input all necessary data into the program to generate an accurate strut adjustment treatment plan. The patient is burdened by the necessity of managing the frame in their daily lives as well as correctly making the daily strut adjustments to achieve the surgical objectives. Errors in calculating both deformity and mounting parameters and other factors in the execution of the plans may result in small but clinically significant residual deformities at the end of treatment which requires the surgeon to iterate the process again in what is called a “re-plan” to bring the residual deformity into an acceptable range [2, 15, 17–20].

Existing published studies on HRF systems are small as their use is not frequent. As a result, most hospital systems, individual surgeons or surgeon teams may not gather large enough populations of patients for clinical evaluations of risks and outcomes [10, 15, 16, 21, 22]. Studies describing outcomes, complications, healthcare resource utilization (HCRU), and costs associated with HRF systems are scarce. This study focuses on the intra-operative period when HRF systems are first applied to the patient (defined herein as the “index”), and is designed to describe the pathology, diagnosis, comorbidities, complications, costs, healthcare utilization and hospital discharge disposition of patients with HRF application during these early intraoperative time points. The post-operative outcomes, from index to 2-year post-application, are described in a separate publication [23].

## Methods

### Data source

This retrospective study identified patients undergoing HRF application in the Premier Hospital Perspective™ Billing Database between 2007 and 2019. The Premier Perspective™ hospital database contains hospital discharge and billing records from over 1000 hospitals across the US. The database was developed for measuring quality and use of health care resources. Participating hospitals represent all regions of the US and are predominantly small-to-mid size nonteaching facilities that serve largely urban patient populations. Hospitals within the Premier Perspective Database are self-selected. They pay a fee and are on contract with Premier to receive access to informatics tools and services. The hospitals submit data voluntarily, and their primary hospital characteristics are representative of those within the American Hospital Association’s annual survey of hospitals in the United States. Unlike individual insurance databases, data within the Premier Perspective Database are not limited by payer status. Available data include admission and discharge characteristics, hospital characteristics, billing information, patient demographics, physician information, cost and charge data.

All data in the PREMIER database being de-identified, the use of these data for this study was exempt from Institutional Review Board oversight as dictated by Title 45 Code of Federal Regulations, Part 46 of the United States, specifically 45 CFR 46.101(b)(4). All methods were carried out in accordance with relevant guidelines and regulations. Experimental protocols were drafted and approved by all co-authors and the Medical Devices Epidemiology research team within the Johnson & Johnson, prior to the conduct of the study.

### Patient population

All patients from 2007 to 2019 in the inpatient setting with a common procedural terminology (CPT), international classification of disease (ICD) code, hospital charge master or charge master records indicative of HRF were included. Application of HRF was identified with a CPT code 20,696, defined as application of multiplane fixation with stereotactic computer-assisted adjustment. The date of application of the frame was defined as the “index date”. Data were analyzed for the duration of the patient’s hospitalization. Patients were categorized based on the diagnosis categories associated with HRF treatment. The following categories were developed based on primary and secondary diagnosis codes associated with the index procedure.

1. Congenital deformity: patients less than 17 years of age, with at least one diagnosis of congenital bone deformity, and no other diagnoses of deep bone infection, nonunion or fracture.
2. Complex congenital deformity: patients less than 17 years of age, with congenital deformity, and concurrent diagnoses of fracture or infection or nonunion.
3. Acquired deformity: patients 17 years or older, with a diagnosis of deformity and no diagnoses of deep infection or nonunion or fracture or arthropathy.

The following categories included patients of all ages but pediatric (defined as < 17) and adult (defined as ≥ 17) patients were analyzed separately:

4. Fracture: patients with a diagnosis of acute fracture and no concurrent diagnosis of deep infection or nonunion or other sequelae, suggesting prior unresolved fracture pathologies.
5. Deep infection, with or without nonunion: patients with deep infection diagnoses (osteomyelitis or infection due to internal fixation or pyogenic arthritis), with or without nonunion diagnoses. Pediatric vs adult patients were further analyzed separately. Pediatric patients with deep infection and congenital deformity were categorized as complex congenital deformity cases, as described above.
6. Non-union without deep infection included patients with non-union diagnoses but no deep infection diagnoses.
7. Arthropathy: Patients with a diagnosis of arthropathy and none of the other diagnoses listed above (deformity, fracture, infection, or non-union) were included in this cohort.

Patients that did not meet any of the defined categories were excluded, as the exact cause for the use of the HRF could not be determined. In the per-category analyses: groups containing less than 30 patients were not analyzed separately as statistical analyses were not meaningful in such small sample sizes.

### Study measures

#### Baseline demographic and clinical characteristics

Patient demographic and clinical characteristics that were evaluated included age, gender, race, census region, marital status, admission source (emergency vs elective), and payer. Baseline comorbidity (i.e., comorbid conditions present on admission) was assessed using the Elixhauser Comorbidity Index, an aggregate measure of comorbidity created by using 31 dimensions associated with chronic disease and overall health conditions. Higher values on Elixhauser are associated with greater comorbidity. Prior research has shown that Elixhauser scores are associated with risk of mortality [24]. Comorbidities analyzed using the Elixhauser index include for example cardiological conditions (congestive heart failure, cardiac arrythmia), diabetes and hypertension (with or without chronic complications), and chronic pulmonary disease, which is defined as all chronic lower respiratory diseases (including asthma) and lung diseases due to external agents. A complete list of all 31 conditions and associated definitions has been previously published [25].

#### Peri-operative complications, costs, and discharge status

Length of inpatient hospital stay (LOS), operating room time (ORT), peri-operative complications and discharge status were evaluated. Post-surgical complications were analyzed for each patient. These complications represent typical post-operative diagnoses, that may be due to a surgical intervention, and are not specific to orthopedic surgery. To be listed as a complication, diagnoses could not be present on admission but had to be given during the hospital stay. These complications included: acute renal failure, bleeding, device failure, wound disruption, dumping, delirium, dysphagia, dysrhythmia, fistula, heart failure, infection (unspecified postoperative, central venous catheter, pneumonia, sepsis, urinary tract), myocardial infarction, nausea/vomiting, shock/body reaction to implant, respiratory failure, retained foreign body, seroma, stroke, subcutaneous emphysema, other surgical injury (accidental puncture and laceration), thrombophlebitis/DVT, transient ischemic attack (TIA) and reaction to transfusions. A list of all ICD-9 and ICD-10 diagnosis codes is included in the Appendix within the Supplemental Files. Hospital costs were adjusted for inflation to consumer price-index of 2020 and calculated for all patients.

### Statistical analyses

All study variables were analyzed descriptively. Counts and proportions (dichotomous variables) and mean and standard deviation (continuous variables) were provided. Estimate of hospital cost and ORT adjusted for age, comorbidity and gender – for each diagnosis category of patients (with N > 30) – were generated using generalized linear models with log link function and gamma distribution. For payment estimates: analyses were only performed on patients with reported index payments > $1000 (to eliminate cases with missing reported payments). For LOS and counts of complications were estimated using Poisson models with log links, adjusting for age, gender and comorbidity. Two multivariable logistic regression models were built to evaluate risk factors for at least one, or at least two, intraoperative complications. For these models, variable selection was performed using a stepwise regression (R package: MASS – function stepAIC). All statistical analyses were performed in R (version 4.0.3) using the RStudio interface (version 1.4.1103).

## Results

A total of 1,867 patients had codes indicative of HRF in the Premier Hospital dataset from 2007 to 2019. Fifteen patients had a length of stay > 100 days or a cost ≥ US$300 K and represented the top 1%. These patients – representing the outliers – are very important from a healthcare and payer standpoint but they were excluded from this analysis as their care cannot be compared to the average patient. An additional 34 patients could not be categorized based on the diagnosis categories defined above. Since the reason for their treatment could not be ascertained, they were also removed from this analysis. The total population therefore included 1,818 patients.

### Baseline demographic and clinical characteristics

Patient demographics and clinical characteristics are shown in Table 1.

**Table 1:** Baseline demographics, admission type and comorbidity index of patients with HRF

Variables	Overall		Patient Group			
			**Younger than 17**		**17 and Above**	
	**N**	**%**	**N**	**%**	**N**	**%**
**All**	**1,818**		**130**		**1,688**	
**Female**	707	38.9%	49	37.7%	658	39.0%
**Age (mean (standard deviation))**	46.9 (19.6)		10.6 (4.2)		49.7 (17.4)	
**Age category**						
Less than 17	130	7.2%	130	100.0%		
17 to 25	176	9.7%			176	10.4%
26 to 45	513	28.2%			513	30.4%
46 to 64	661	36.4%			661	39.2%
65 and Above	338	18.6%			338	20.0%
**Race**						
Black	286	15.7%	40	30.8%	246	14.6%
White	1,227	67.5%	65	50.0%	1,162	68.8%
Hispanic	35	1.9%	3	2.3%	32	1.9%
Other or Unavailable	270	14.9%	22	16.9%	248	14.7%
**Payer**						
Commercial	727	40.0%	65	50.0%	662	39.2%
Medicaid	304	16.7%	47	36.2%	257	15.2%
Medicare	418	23.0%	1	0.8%	417	24.7%
Other	369	20.3%	17	13.1%	352	20.9%
**Admission**						
Emergency or Urgent Care	917	50.4%	28	21.5%	889	52.7%
Trauma Center	202	11.1%	3	2.3%	199	11.8%
**Etiology**						
Congenital deformity without fractures	75	4.1%	75	57.7%		
Congenital deformity with fracture or sequelas from prior fracture	4	0.2%	4	3.1%		
Deep Infection without nonunion	122	6.7%	1	0.8%	121	7.2%
Deep infection with non-union	52	2.9%	1	0.8%	51	3.0%
Fracture	1,319	72.6%	44	33.8%	1,275	75.5%
Non-Union	76	4.2%	3	2.3%	73	4.3%
Other Sequelas from Prior Fractures	26	1.4%	2	1.5%	24	1.4%
Acquired Deformity	107	5.9%			107	6.3%
Arthrosis	37	2.0%			37	2.2%
**Average Elixhauser Score (mean (standard deviation))**	1.90 (2.16)		0.45 (0.84)		2.02 (2.19)	

The mean (SD) age of patients was 46.9 (19.6) years and 38.9% (707/1,818) of patients were female. Fracture was the most common etiology (72.6%—1,319/1,818), followed by deep infection (without nonunion: 6.7% (122/1,818), with nonunion: 2.9% (52/1,818)) and deformity (acquired deformity: 5.9% (107/1,818), congenital deformity 4.1% (75/1,818)). More than half of all adult admissions were emergency cases (889/1,688), whereas only 21.5% (28/130) pediatric cases were emergency cases.

Table 2 and 3 outlines the observed comorbidities in all cohorts.

**Table 2:** Key baseline comorbidities at time of HRF application. 2: By age category; Table 3: By diagnosis category. The categories: “Deep Infection with Nonunion” and “Deep Infection Only” were aggregated under “Deep Infection” as values were similar for both. Comorbidity by Patient Age Group

Variables	Overall (*N* = 1,818)	Adult (*N* = 1,688)	Pediatric (*N* = 130)
Hypertension (with or without complications)	737 (40.5%)	736 (43.6%)	1 (0.8%)
All Diabetes (with or without complications)	432 (23.8%)	431 (25.5%)	1 (0.8%)
*Complicated Diabetes*	233 (12.8%)	233 (13.8%)	0 (0.0%)
Obesity	276 (15.2%)	255 (15.1%)	21 (16.2%)
Chronic Pulmonary Diseases (including asthma)	273 (15.0%)	259 (15.3%)	14 (10.8%)
Fluid and Electrolyte Disorders	225 (12.4%)	223 (13.2%)	2 (1.5%)
Depression	217 (11.9%)	214 (12.7%)	3 (2.3%)
Cardiac Arrythmia	153 (8.4%)	151 (8.9%)	2 (1.5%)
Hypothyroidism	149 (8.2%)	148 (8.8%)	1 (0.8%)
Alcohol Abuse	141 (7.8%)	140 (8.3%)	1 (0.8%)
Renal Failure	139 (7.6%)	139 (8.2%)	0 (0.0%)
Drug Abuse	126 (6.9%)	124 (7.3%)	2 (1.5%)

**Table 3:** Key baseline comorbidities at time of HRF application. Table 2: By age category; Table 3: By diagnosis category. The categories: “Deep Infection with Nonunion” and “Deep Infection Only” were aggregated under “Deep Infection” as values were similar for both. Comorbidity by Diagnosis Group—Adult Population

	Adult Patients					Pediatric Patients	
Variables	**Acquired Deformity (*N* = 107)**	**Arthrosis (*N* = 37)**	**Deep Infection, with or without nonunion (*N* = 172)**	**Fracture (*N* = 1,275)**	**Nonunion—no infection (*N* = 73)**	**Congenital Deformity (*N* = 75)**	**Fracture (*N* = 44)**
Age (mean, standard deviation)	**44.4 (17.2)**	**54.7 (10.6)**	**51.7 (16.6)**	**49.7 (17.7)**	**48.7 (16.3)**	**9.7 (4.4)**	**12.2 (3.6)**
All Hypertension (with or without complications)	47 (43.9%)	25 (67.6%)	106 (61.6%)	506 (39.7%)	35 (47.9%)	1 (1.3%)	0 (0.0%)
All Diabetes (with or without complications)	42 (39.3%)	29 (78.4%)	89 (51.7%)	239 (18.7%)	16 (21.9%)	1 (1.3%)	0 (0.0%)
*Complicated Diabetes*	28 (26.2%)	17 (45.9%)	59 (34.3%)	112 (8.8%)	7 (9.6%)	0 (0.0%)	0 (0.0%)
Obesity	27 (25.2%)	7 (18.9%)	27 (15.7%)	174 (13.6%)	12 (16.4%)	16 (21.3%)	4 (9.1%)
Chronic Pulmonary Disease	12 (11.2%)	4 (10.8%)	33 (19.2%)	195 (15.3%)	11 (15.1%)	7 (9.3%)	6 (13.6%)
Fluid and Electrolyte Disorders	7 (6.5%)	4 (10.8%)	37 (21.5%)	164 (12.9%)	8 (11.0%)	1 (1.3%)	1 (2.3%)
Depression	12 (11.2%)	5 (13.5%)	33 (19.2%)	146 (11.5%)	14 (19.2%)	0 (0.0%)	2 (4.5%)
Cardiac Arrythmia	6 (5.6%)	3 (8.1%)	27 (15.7%)	107 (8.4%)	2 (2.7%)	1 (1.3%)	1 (2.3%)
Hypothyroidism	12 (11.2%)	4 (10.8%)	33 (19.2%)	105 (8.2%)	4 (5.5%)	1 (1.3%)	0 (0.0%)
Alcohol Abuse	1 (0.9%)	2 (5.4%)	13 (7.6%)	121 (9.5%)	2 (2.7%)	0 (0.0%)	1 (2.3%)
Renal Failure	10 (9.3%)	6 (16.2%)	25 (14.5%)	84 (6.6%)	6 (8.2%)	0 (0.0%)	0 (0.0%)
Drug Abuse	0 (0.0%)	0 (0.0%)	18 (10.5%)	103 (8.1%)	3 (4.1%)	0 (0.0%)	2 (4.5%)
Average Elixhauser Index	**1.9 (1.9)**	**2.7 (1.9)**	**3.0 (2.3)**	**1.8 (2.1)**	**1.8 (2.1)**	**0.5 (0.8)**	**0.4 (1.0)**

As shown in Table 2, Obesity and pulmonary disease/asthma were the most prevalent comorbidities in children (16.2% (21/130) and 10.8% (14/130), respectively), followed by depression (2.3% (3/130)), cardiac arrythmia and fluid, electrolyte disorders and drug abuse (1.5% in all 3 cases (2/130)). In the adult population, there were 43.6% patients with hypertension (736/1,688), 25.6% (431/1,688) with diabetes (13.8% (233/1,688) had complicated diabetes), 15.1% (255/1,688) obesity, 15.3% (259/1,688) chronic pulmonary diseases/asthma, 13.2% (223/1,688) fluid and electrolyte disorders and 12.7% (214/1,688) depression. A closer analysis by diagnosis category further highlights differences across groups, as shown in Table 3. This table also includes average age by group, as many of the comorbidities may be result of increasing age in the group. Patients presenting with arthrosis or deep infection had the greatest percentage of hypertension and diabetes and had also the highest average age (average age: 54.7 (SD: 10.6) and 51.7 (SD: 16.6), respectively versus less than 50 for all other categories). Complicated diabetes was present in nearly half of all arthropathy patients (45.9% (17/37)) and a third of deep infection patients (34.3% (59/172)). In contrast, none of the pediatric cases had complicated diabetes.

Admission for care also differed by group: 98/107 adult deformity cases (91.6%) were treated electively, and 67/75 congenital deformity cases (89.3%) were elective. More than 50% of deep infection cases were elective procedure (58/122 deep infection without nonunion, and 28/52 deep infection with nonunion) whereas only 257/1,319 (19.5%) fracture cases were elective.

#### Operative healthcare utilization

The ORT, LOS and inflation-adjusted hospital costs for all patients averaged 277.7 min (SD: 273.6), 7.07 days (SD 9.4) and $41,507 (SD 38,694), respectively. Model outputs for ORT, LOS and costs, by diagnosis category, are shown in Table 4.

**Table 4:** Operating room time (ORT - in minutes), length of hospital stay (LOS - in days), and inflation-adjusted total hospital cost for index admission for patients treated with HRF, by diagnosis category. The categories: “Deep Infection with Nonunion” and “Deep Infection Only” were aggregated under “Deep Infection” as values were similar for both

Index Diagnosis Category	Operating Room Time (minutes)	Length of Hospital Stay (days)	Index Cost
Adult – Acquired Deformity	228.1 (193.1–278.8)	2.9 (2.5–3.3)	$38,217 ($32,105-$47,204)
Adult – Arthropathy	213.5 (159.9–321.2)	4.3 (3.5–5.3)	$37,084 ($27,907-$55,253)
Adult—Deep Infection	369.0 (321.0–433.8)	14.4 (13.7–15.1)	$54,666 ($47,960-$63,553)
Adult – Fracture	273.0 (259.0–288.5)	9.5 (9.2–9.7)	$40,273 ($38,239-$42,535)
Adult – Non Union (no infection)	262.5 (214.0–339.5)	7.4 (6.5–8.3)	$40,537 ($32,948-$52,669)
Pediatric – Congenital Deformity	205.3 (173.1–252.3)	4.6 (4.0–5.4)	$37,588 ($30,852-$48,088)
Pediatric – Fracture	183.1 (148.1–239.7)	6.1 (5.3–7.0)	$30,384 ($24,153-$40,947)

Patients with deep infection had the longest overall ORT (approximately 6 h) over an average 14.4 day LOS. Their average index cost amounted to approximately $54.6 K.

**Fig. 1:**
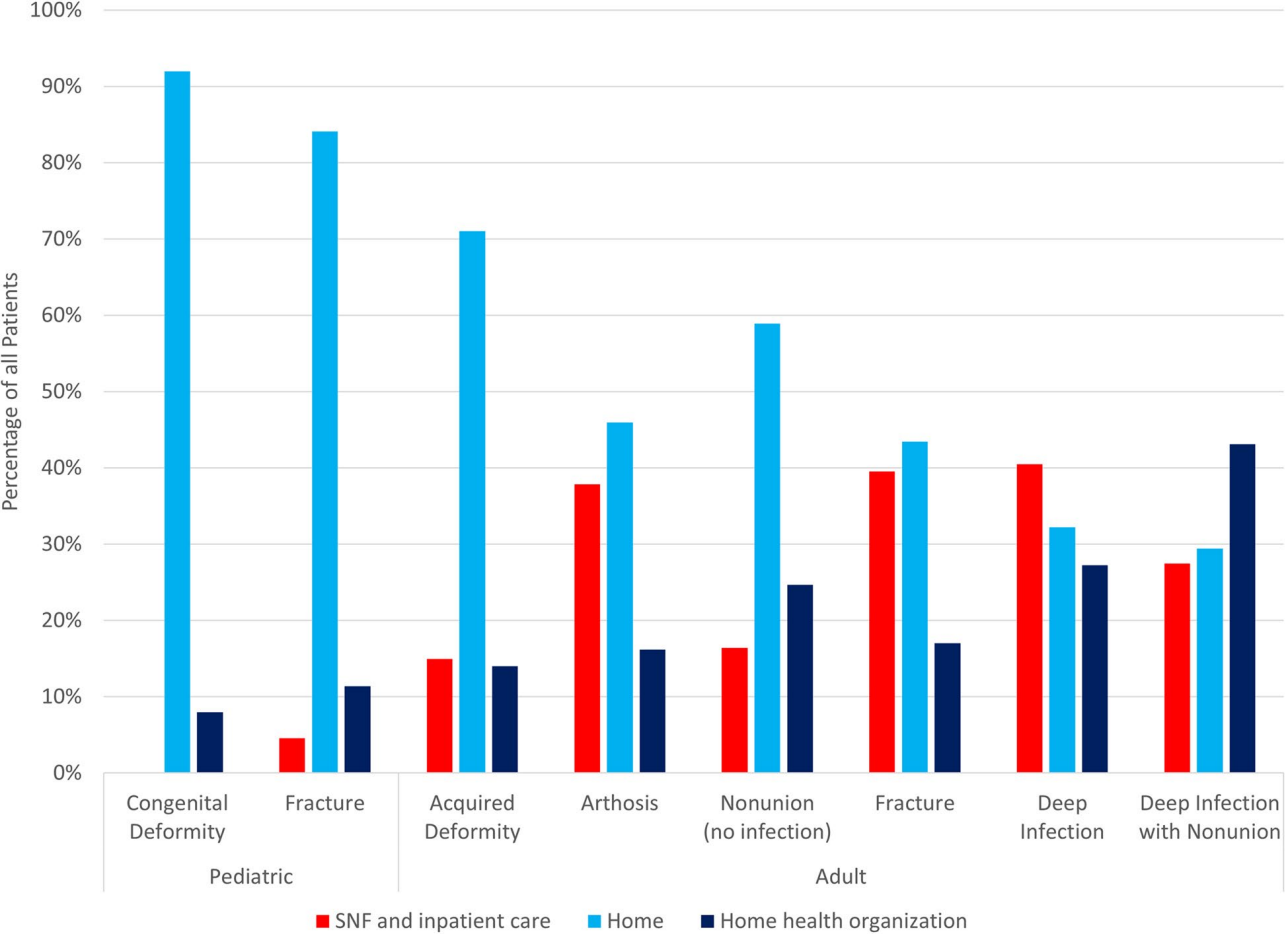
Discharge disposition of patients treated with HRF. Pediatric patients treated for deformity were all discharged to home (92%) or home health (8%). Adult patients treated for deep infection with nonunion had only a 29% rate of home discharge

Discharge status is shown for all patients in Fig. 1. All pediatric patients treated for congenital deformity were discharged to either home (69/75—92%) or home health (6/75—8%). Patients with deep infection with nonunion had the lowest rate of home discharge, with 15 out of 51 patients (29%) discharged home, 22/51 (43%) to home health and 14/51 (27%) to a skilled nursing facility (SNF) or inpatient care.

#### Intraoperative complications

A total of 24% patients had at least one intraoperative complication, and 8% had two or more complications. The main intraoperative complication types are shown in Table 5. Bleeding was the most common complication, affecting 11.8% of all patients. Most patients with intraoperative bleeding were adults (12.6% of all adults), only 1.6% pediatric cases experienced.

**Table 5:** Intraoperative complications by age category. Intraoperative complications were rare in pediatric cases. Key Complication types by age group

Complication Types	Overall	Adult	Pediatric
Complication Bleeding	11.8%	12.6%	1.6%
Respiratory Failure	4.2%	4.4%	2.3%
Dysrhythmias	3.4%	3.7%	0%
Infection-Pneumonia/Pneumonitis	2.9%	3.1%	0.8%
Acute Renal Failure	2.7%	3.0%	0%
Infection and/or Sepsis	1.6%	1.8%	0%
Heart Failure	1.4%	1.5%	0%
Infection-Urinary Tract	1.4%	1.5%	0%
Complication Disruption of wound	0.9%	0.9%	0%
Nausea/Vomiting	0.9%	0.8%	1.5%
Dysphagia	0.7%	0.7%	0.8%

bleeding. Overall, intraoperative complications were rare in pediatrics compared to adults. The probability of experiencing at least one- or at least two complications are shown in Table 6.

**Table 6:** Intraoperative complications by age category. Intraoperative complications were rare in pediatric cases. Count of complications and probability of experiencing at least one- or at least two-complications during the admission. The diagnostic categories: “Deep Infection with Nonunion” and “Deep Infection Only” were aggregated as values were similar for both. (None of the patients in the diagnostic categories: Adult – Arthropathy or Pediatric – Congenital Deformity had two or more complications in our dataset.)

Complication Count and Probability By Diagnosis Category	Average Count of Complications	Probability of ≥ One Complication	Probability of ≥ Two Complications
Adult – Acquired Deformity	0.2 (0.1–0.3)	14% (8%-22%)	4% (2%-10%)
Adult – Arthropathy	0.1 (0.1–0.3)	14% (6%-28%)	
Adult—Deep Infection	0.5 (0.4–0.6)	35% (28%-43%)	10% (7%-16%)
Adult – Fracture	0.3 (0.3–0.4)	23% (21%-26%)	7% (5%-8%)
Adult – Non Union (no infection)	0.3 (0.2–0.4)	18% (10%-30%)	5% (2%-15%)
Pediatric – Congenital Deformity	0.1 (0.0–0.2)	7% (2%-20%)	
Pediatric – Fracture	0.2 (0.1–0.4)	15% (6%-32%)	3% (0%-21%)

**Fig. 2:**
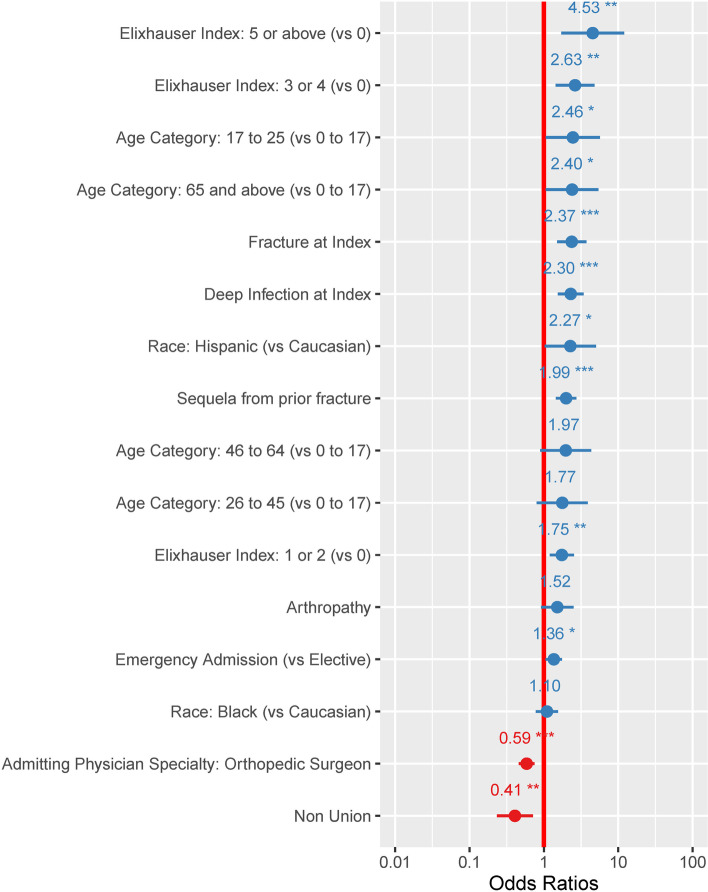
Odds ratios of variables associated with having at least one perioperative complication during the admission when the HRF was applied. Presence of multiple chronic comorbidities were predictive for postoperative complications. Patients with 5 or more comorbid conditions (Elixhauser 5 or above) had an odds ratio of 4.53 (95%CI: 1.70–12.01). Patients with factures and deep infection were also at greater risk of complications (OR: 2.37 (95%CI: 1.51–3.72) and OR: 2.30 (95%CI: 1.54–3.42), respectively). *: *p* value < 0.05; **: *p* value < 0.005; ***: *p* value < 0.0001

**Fig. 3:**
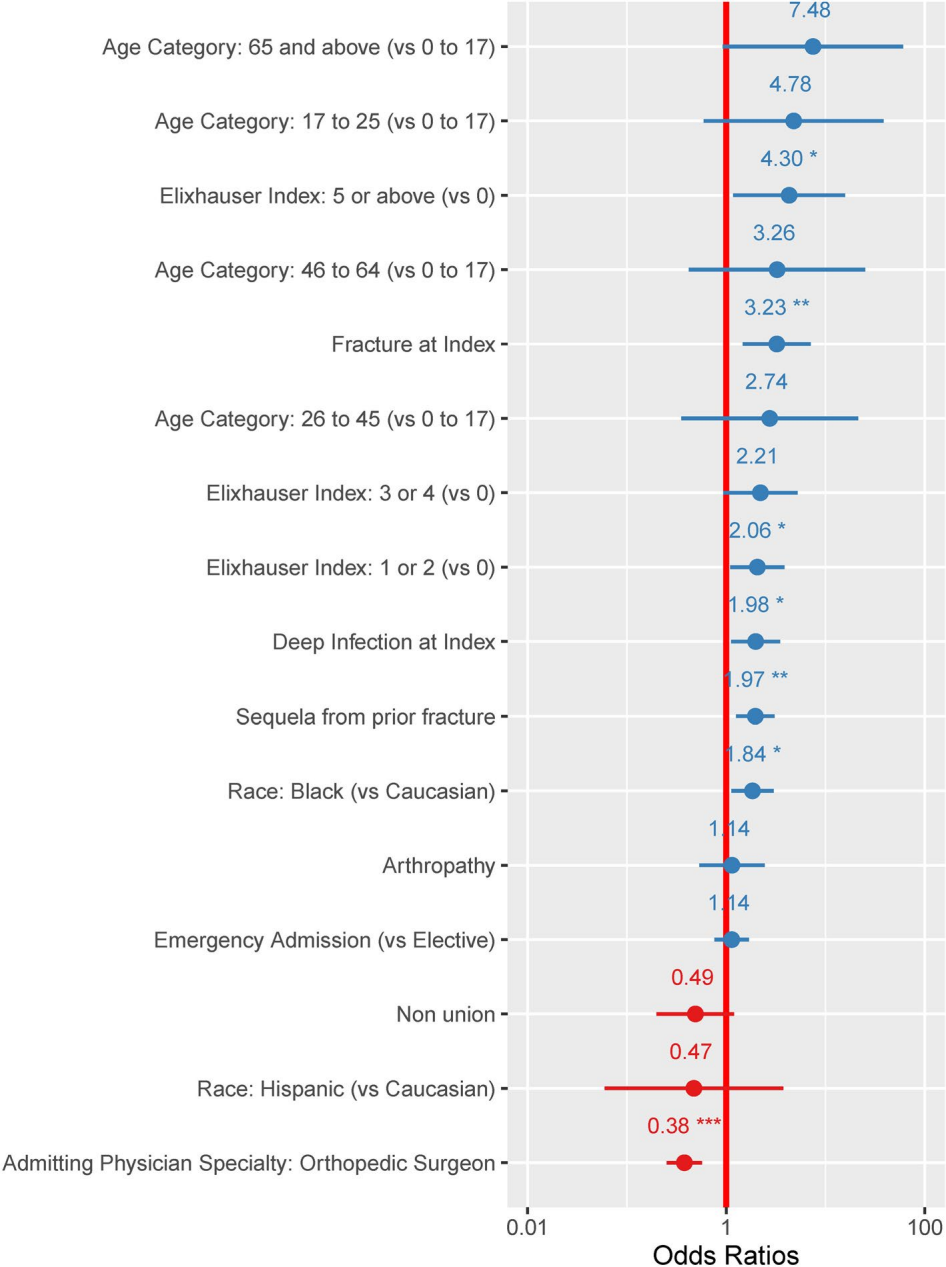
Odds ratios of variables associated with having at least two perioperative complications during the admission when the HRF was applied. Presence of multiple chronic comorbidities were predictive for postoperative complications. Patients with 5 or more comorbid conditions (Elixhauser 5 or above) had an odds ratio of 4.30 (95%CI: 1.18–15.71). In addition to complications, fracture as diagnosis category was also associated with two or more intraoperative complications (OR: 3.23 (95%CI: 1.47–7.11)). *: *p* value < 0.05; **: *p* value < 0.005; ***: *p* value < 0.0001

Patients treated for congenital deformity had an average of 7% (95%CI: 2%-20%) probability of experiencing one complication. None of the patients treated for congenital deformity in our cohort experienced two or more complications. Pediatric patients treated for fractures had slightly higher complication probabilities, averaging 15% (95%CI: 6%-32%) for one complication and 3% (95%CI: 0%-21%) for two or more complications. Logistic regression models looking at risk for one or two- or more complications showed that pre-existing comorbidities, especially in patients with multiple concurrent comorbidities (Elixhauser 5 or above) were significantly associated with risks of intraoperative complications, as shown in Fig. 2 and 3. Fracture, and deep infection, were also independently associated with higher risks of intraoperative complications. In contrast, admissions where orthopedic surgeons were the admitting and treating physicians were less likely to be associated with intraoperative complications.

## Discussion

Our study including 1,818 patients from > 1000 hospitals across the US identified the heterogeneity in etiology and risk among patients being treated with an HRF application. There were large differences between diagnosis categories in the duration of surgery, length of hospitalization, peri-operative complications, hospital costs, and discharge status; This fact is particularly important as it highlights the diverse use of HRF. Outcomes related to HRF are highly dependent on the population (adult versus pediatric) and diagnosis of the patients for which HRF is used. Unlike other devices with more narrow indications, clinical findings from HRF research are not generalizable beyond to the exact patient populations for which HRF are studied. Pooling of data from HRF patients across age and etiology is also problematic as these patients have such different clinical presentations and risks, as shown in our study. Our study differs from other published evidence as it includes a relatively large sample size of patients with very different clinical presentations, all treated with HRF exclusively. Comparatively, other published studies focused on smaller cohorts, with specific diagnoses or treated with a mix of external fixators [Tafanel et al.*:* 25 pediatric fracture cases, [10] Shore et al.*:* 44 fracture cases, [21] Fenton et al.*:* 21 nonunion cases treated with any external fixator, [16] Feldman et al.*:* 18 nonunion cases [22]]. One of the largest published studies describes outcomes in 102 pediatric and adult patients, all presenting for deformity correction [15]. Published studies also focused on the post-operative, post-discharge period, whereas we focused on understanding differences between patients at time of HRF application.

In our study, patients with congenital or acquired deformities had few (if any) intra-operative complications and were more likely to be discharged home compared to all other patients, probably due to the elective nature of their procedures and the fact that these patients were generally healthy pre-index. Deep infection patients, as expected, were the most complex and costly.

The most common peri-operative complications observed in the HRF patients were bleeding and respiratory failure. These complications were based solely on presence of diagnosis codes, not on added procedures or interventions. These complications are typical of long procedures, regardless of implant use. Rates of intraoperative complications were very low for elective deformity correction cases, whereas they were high for fracture cases and complex deep infection cases, which were also associated with longer OR times. In addition, there was a strong association between presence of multiple comorbidities at time of admission, as indicated by an Elixhauser score > 5, and complication rates, which suggests that intraoperative complications were mostly driven by the overall health status of the patients rather than the HRF application procedure. Patients that presented with non-union without infection had lower overall comorbidities vs patients with infection (as seen on Table 3: 1.8 Elixhauser index vs 3.0 Elixhauser index for patients with deep infection, and 9.6% complicated diabetes versus 34.3% in the deep infection category). They were also found to be at lower risk for complications. Their lower rates of complications may therefore be due to their overall better health. Another interesting finding was the fact that admissions led by orthopedic surgeons were at lower risk of complications. This may be due to orthopedic surgeon-led cases being more likely elective or within larger trauma centers. Interestingly, being an “emergency” case was not associated with increased intra/post operative complications. 

In this narrow, perioperative time period, patients with deep infection with nonunions were not different from patients with deep infection without nonunions, when analyzing adjusted means for costs or complications; however, in our second manuscript looking at the 2-year post-index period, patients with deep infection and nonunion did have greater post-operative care requirements and complication risks in the time frame from day after surgery to two years post-index [23].

A key strength of the database (Premier) used for this analysis is that it captures a broad representation of patients across the US, including patients with different healthcare insurance; however, this is also a limitation of our study, as variables available in the database were not prospectively collected for research purposes. In addition, the Premier database may not include large academic trauma centers that specialize in the application of HRF for complex problems and thus may omit an important patient group. Surgeon notes and details of surgical approaches not captured in standard codes (CPT or ICD) and patient-reported outcomes are not captured in this dataset. Large databases such as Premier are also at risk of having clerical inaccuracies, recording bias secondary to financial incentives, and temporal changes in billing codes [26, 27]. Additional limitations includes the facts that: 1) there was no adjustment for regional differences across the US; 2) this study aggregates 12 years of data, during which changes of healthcare delivery may have occurred, and 3) adjustment for surgeon- or hospital-specific care patterns were not made. Despite these limitations, this study provides an informative overview of the experience of care that patients had in the hospital upon the initial application of a HRF.

## Conclusions

Peri-operative outcomes and risks during HRF varied based on patient pathology and diagnosis and were mostly associated with age and comorbidities. Large variability in complication rates, discharge disposition and length of hospital stay describes the heterogeneous nature of patients treated with HRF. This study provides a detailed understanding of costs and outcomes in the current HRF population.

## Supplementary Information

**Additional file 1.**

## Data Availability

The data for these analyses were made available to the authors by third-party licenses from PREMIER (https://products.premierinc.com/applied-sciences/solutions/applied-research-and-analytics), a data provider in the US. Under the licensing agreement, the authors cannot provide raw data themselves. Other researchers could access the data by purchase through PREMIER, and the inclusion criteria specified in the Methods section would allow them to identify the same cohort of patients we used for these analyses.

## Abbreviations

CPT: Common Procedural Terminology
HCRU: Health Care Resource Utilization
HRF: Hexapod Ring Fixation
ICD: International Classification of Disease
LOS: Length of Stay
ORT: Operating Room Time
SD: Standard Deviation
SNF: Skilled Nursing Facility
