# Supplementary material for: Hospitalization for computer-assisted hexapod ring fixation application – analyses of patient variability, peri-operative complications, hospital costs, and discharge status
Source: BMC Musculoskelet Disord. 2022 Mar 5;23:211. doi: 10.1186/s12891-022-05171-6 (PMC8897910; doi:10.1186/s12891-022-05171-6)
Supplement: Supplementary file 1 — Additional file 1. [file 12891_2022_5171_MOESM1_ESM.docx]

**Supplemental Tables**

1. Logistic Regression outputs: Risk of Experiencing at least 1 complication

1. Logistic Regression outputs: Risk of Experiencing at least 2 complications

**APPENDIX**: Code list for Postoperative Complications

| Complication | ICD Classification | Code | Short Description |
| --- | --- | --- | --- |
| Acute Renal Failure | 9 | 584.5 | Acute kidney failure with lesion of tubular necrosis |
| Acute Renal Failure | 9 | 584.6 | Acute kidney failure with lesion of renal cortical necrosis |
| Acute Renal Failure | 9 | 584.9 | Acute kidney failure unspecified |
| Acute Renal Failure | 10 | N170 | Acute kidney failure with tubular necrosis |
| Acute Renal Failure | 10 | N171 | Acute kidney failure with acute cortical necrosis |
| Acute Renal Failure | 10 | N179 | Acute kidney failure unspecified |
| Bleeding | 9 | 285.1 | Acute posthemorrhagic anemia |
| Bleeding | 9 | 459 | Hemorrhage unspecified |
| Bleeding | 9 | 998.11 | Hemorrhage complicating a procedure |
| Bleeding | 9 | 998.12 | Hematoma complicating a procedure |
| Bleeding | 10 | D62 | Acute posthemorrhagic anemia |
| Bleeding | 10 | D7822 | Postprocedural hemorrhage and hematoma of the spleen following other procedure |
| Bleeding | 10 | G9732 | Intraoperative hemorrhage and hematoma of a nervous system organ or structure complicating other procedure |
| Bleeding | 10 | G9751 | Postprocedural hemorrhage and hematoma of a nervous system organ or structure following a nervous system procedure |
| Bleeding | 10 | G9752 | Postprocedural hemorrhage and hematoma of a nervous system organ or structure following other procedure |
| Bleeding | 10 | I9742 | Intraoperative hemorrhage and hematoma of a circulatory system organ or structure complicating other procedure |
| Bleeding | 10 | M96810 | Intraoperative hemorrhage and hematoma of a musculoskeletal structure complicating a musculoskeletal system procedure |
| Bleeding | 10 | M96811 | Intraoperative hemorrhage and hematoma of a musculoskeletal structure complicating other procedure |
| Bleeding | 10 | M96830 | Postprocedural hemorrhage and hematoma of a musculoskeletal structure following a musculoskeletal system procedure |
| Bleeding | 10 | M96831 | Postprocedural hemorrhage and hematoma of a musculoskeletal structure following other procedure |
| Bleeding | 10 | R58 | Hemorrhage not elsewhere classified |
| Delirium | 9 | 292.81 | Drug-induced delirium |
| Delirium | 9 | 293 | Delirium due to conditions classified elsewhere |
| Delirium | 10 | F05 | Delirium due to known physiological condition |
| Delirium | 10 | F19921 | Other psychoactive substance use unspecified with intoxication with delirium |
| Device Failure | 9 | 996.59 | Mechanical complication due to other implant and internal device not elsewhere classfied |
| Device Failure | 9 | 996.7 | Other complications due to unspecified device implant and graft |
| Device Failure | 10 | T85692A | Other mechanical complication of permanent sutures initial encounter |
| Device Failure | 10 | T85698A | Other mechanical complication of other specified internal prosthetic devices implants and grafts initial encounter |
| Device Failure | 10 | T859XXA | Unspecified complication of internal prosthetic device implant and graft initial encounter |
| Disruption of wound | 9 | 998.3 | Disruption of wound unspecified |
| Disruption of wound | 9 | 998.31 | Disruption of internal operation - surgical- wound |
| Disruption of wound | 9 | 998.32 | Disruption of external operation - surgical- wound |
| Disruption of wound | 9 | 998.83 | Non-healing surgical wound |
| Disruption of wound | 10 | T8130XA | Disruption of wound unspecified initial encounter |
| Disruption of wound | 10 | T8131XA | Disruption of external operation (surgical) wound not elsewhere classified initial encounter |
| Disruption of wound | 10 | T8132XA | Disruption of internal operation (surgical) wound not elsewhere classified initial encounter |
| Dumping | 9 | 579.3 | Other and unspecified postsurgical nonabsorption |
| Dumping | 10 | K912 | Postsurgical malabsorption not elsewhere classified |
| Dysphagia | 9 | 787.2 | Dysphagia unspecified |
| Dysphagia | 9 | 787.29 | Other dysphagia |
| Dysphagia | 10 | R130 | Aphagia |
| Dysphagia | 10 | R1310 | Dysphagia unspecified |
| Dysphagia | 10 | R1319 | Other dysphagia |
| Dysrhythmias | 9 | 427 | Paroxysmal supraventricular tachycardia |
| Dysrhythmias | 9 | 427.1 | Paroxysmal ventricular tachycardia |
| Dysrhythmias | 9 | 427.2 | Paroxysmal tachycardia unspecified |
| Dysrhythmias | 9 | 427.31 | Atrial fibrillation |
| Dysrhythmias | 9 | 427.32 | Atrial flutter |
| Dysrhythmias | 9 | 427.41 | Ventricular fibrillation |
| Dysrhythmias | 9 | 427.42 | Ventricular flutter |
| Dysrhythmias | 9 | 427.5 | Cardiac arrest |
| Dysrhythmias | 9 | 427.6 | Premature beats unspecified |
| Dysrhythmias | 9 | 427.61 | Supraventricular premature beats |
| Dysrhythmias | 9 | 427.69 | Other premature beats |
| Dysrhythmias | 9 | 427.81 | Sinoatrial node dysfunction |
| Dysrhythmias | 9 | 427.89 | Other specified cardiac dysrhythmias |
| Dysrhythmias | 9 | 427.9 | Cardiac dysrhythmia unspecified |
| Dysrhythmias | 10 | I469 | Cardiac arrest cause unspecified |
| Dysrhythmias | 10 | I471 | Supraventricular tachycardia |
| Dysrhythmias | 10 | I472 | Ventricular tachycardia |
| Dysrhythmias | 10 | I479 | Paroxysmal tachycardia unspecified |
| Dysrhythmias | 10 | I4891 | Unspecified atrial fibrillation |
| Dysrhythmias | 10 | I4892 | Unspecified atrial flutter |
| Dysrhythmias | 10 | I4901 | Ventricular fibrillation |
| Dysrhythmias | 10 | I4902 | Ventricular flutter |
| Dysrhythmias | 10 | I491 | Atrial premature depolarization |
| Dysrhythmias | 10 | I493 | Ventricular premature depolarization |
| Dysrhythmias | 10 | I4940 | Unspecified premature depolarization |
| Dysrhythmias | 10 | I4949 | Other premature depolarization |
| Dysrhythmias | 10 | I495 | Sick sinus syndrome |
| Dysrhythmias | 10 | I498 | Other specified cardiac arrhythmias |
| Dysrhythmias | 10 | I499 | Cardiac arrhythmia unspecified |
| Dysrhythmias | 10 | R001 | Bradycardia unspecified |
| Fistula | 9 | 998.6 | Persistent postoperative fistula |
| Fistula | 10 | T8183XA | Persistent postprocedural fistula initial encounter |
| Heart Failure | 9 | 428 | Congestive heart failure unspecified |
| Heart Failure | 9 | 428.1 | Left heart failure |
| Heart Failure | 9 | 428.2 | Systolic heart failure unspecified |
| Heart Failure | 9 | 428.21 | Acute systolic heart failure |
| Heart Failure | 9 | 428.22 | Chronic systolic heart failure |
| Heart Failure | 9 | 428.23 | Acute on chronic systolic heart failure |
| Heart Failure | 9 | 428.3 | Diastolic heart failure unspecified |
| Heart Failure | 9 | 428.31 | Acute diastolic heart failure |
| Heart Failure | 9 | 428.32 | Chronic diastolic heart failure |
| Heart Failure | 9 | 428.33 | Acute on chronic diastolic heart failure |
| Heart Failure | 9 | 428.41 | Acute combined systolic and diastolic heart failure |
| Heart Failure | 9 | 428.42 | Chronic combined systolic and diastolic heart failure |
| Heart Failure | 9 | 428.43 | Acute on chronic combined systolic and diastolic heart failure |
| Heart Failure | 9 | 428.9 | Heart failure unspecified |
| Heart Failure | 10 | I501 | Left ventricular failure |
| Heart Failure | 10 | I5020 | Unspecified systolic (congestive) heart failure |
| Heart Failure | 10 | I5021 | Acute systolic (congestive) heart failure |
| Heart Failure | 10 | I5022 | Chronic systolic (congestive) heart failure |
| Heart Failure | 10 | I5023 | Acute on chronic systolic (congestive) heart failure |
| Heart Failure | 10 | I5030 | Unspecified diastolic (congestive) heart failure |
| Heart Failure | 10 | I5031 | Acute diastolic (congestive) heart failure |
| Heart Failure | 10 | I5032 | Chronic diastolic (congestive) heart failure |
| Heart Failure | 10 | I5033 | Acute on chronic diastolic (congestive) heart failure |
| Heart Failure | 10 | I5041 | Acute combined systolic (congestive) and diastolic (congestive) heart failure |
| Heart Failure | 10 | I5042 | Chronic combined systolic (congestive) and diastolic (congestive) heart failure |
| Heart Failure | 10 | I5043 | Acute on chronic combined systolic (congestive) and diastolic (congestive) heart failure |
| Heart Failure | 10 | I509 | Heart failure unspecified |
| Infection | 9 | 996.6 | Infection and inflammatory reaction due to unspecified device implant and graft |
| Infection | 9 | 998.59 | Other postoperative infection |
| Infection | 10 | K6811 | Postprocedural retroperitoneal abscess |
| Infection | 10 | T814XXA | Infection following a procedure initial encounter |
| Infection | 10 | T8579XA | Infection and inflammatory reaction due to other internal prosthetic devices implants and grafts initial encounter |
| Infection - Central Line Infection | 9 | 999.31 | Other and unspecified infection due to central venous catheter |
| Infection - Central Line Infection | 9 | 999.32 | Bloodstream infection due to central venous catheter |
| Infection - Central Line Infection | 10 | T80211A | Bloodstream infection due to central venous catheter initial encounter |
| Infection - Central Line Infection | 10 | T80219A | Unspecified infection due to central venous catheter initial encounter |
| Infection-Pneumonia/Pneumonitis | 9 | 481 | Pneumococcal pneumonia - Streptococcus pneumoniae pneumonia- |
| Infection-Pneumonia/Pneumonitis | 9 | 482 | Pneumonia due to Klebsiella pneumoniae |
| Infection-Pneumonia/Pneumonitis | 9 | 482.1 | Pneumonia due to Pseudomonas |
| Infection-Pneumonia/Pneumonitis | 9 | 482.2 | Pneumonia due to Hemophilus influenzae - H influenzae- |
| Infection-Pneumonia/Pneumonitis | 9 | 482.39 | Pneumonia due to other Streptococcus |
| Infection-Pneumonia/Pneumonitis | 9 | 482.41 | Methicillin susceptible pneumonia due to Staphylococcus aureus |
| Infection-Pneumonia/Pneumonitis | 9 | 482.42 | Methicillin resistant pneumonia due to Staphylococcus aureus |
| Infection-Pneumonia/Pneumonitis | 9 | 482.89 | Pneumonia due to other specified bacteria |
| Infection-Pneumonia/Pneumonitis | 9 | 482.9 | Bacterial pneumonia unspecified |
| Infection-Pneumonia/Pneumonitis | 9 | 483 | Pneumonia due to mycoplasma pneumoniae |
| Infection-Pneumonia/Pneumonitis | 9 | 483.8 | Pneumonia due to other specified organism |
| Infection-Pneumonia/Pneumonitis | 9 | 485 | Bronchopneumonia organism unspecified |
| Infection-Pneumonia/Pneumonitis | 9 | 486 | Pneumonia organism unspecified |
| Infection-Pneumonia/Pneumonitis | 9 | 507 | Pneumonitis due to inhalation of food or vomitus |
| Infection-Pneumonia/Pneumonitis | 9 | 507.8 | Pneumonitis due to other solids and liquids |
| Infection-Pneumonia/Pneumonitis | 9 | 510 | Empyema with fistula |
| Infection-Pneumonia/Pneumonitis | 9 | 510.9 | Empyema without mention of fistula |
| Infection-Pneumonia/Pneumonitis | 9 | 997.32 | Postprocedural aspiration pneumonia |
| Infection-Pneumonia/Pneumonitis | 10 | J13 | Pneumonia due to Streptococcus pneumoniae |
| Infection-Pneumonia/Pneumonitis | 10 | J14 | Pneumonia due to Hemophilus influenzae |
| Infection-Pneumonia/Pneumonitis | 10 | J150 | Pneumonia due to Klebsiella pneumoniae |
| Infection-Pneumonia/Pneumonitis | 10 | J151 | Pneumonia due to Pseudomonas |
| Infection-Pneumonia/Pneumonitis | 10 | J15211 | Pneumonia due to Methicillin susceptible Staphylococcus aureus |
| Infection-Pneumonia/Pneumonitis | 10 | J15212 | Pneumonia due to Methicillin resistant Staphylococcus aureus |
| Infection-Pneumonia/Pneumonitis | 10 | J154 | Pneumonia due to other streptococci |
| Infection-Pneumonia/Pneumonitis | 10 | J157 | Pneumonia due to Mycoplasma pneumoniae |
| Infection-Pneumonia/Pneumonitis | 10 | J158 | Pneumonia due to other specified bacteria |
| Infection-Pneumonia/Pneumonitis | 10 | J159 | Unspecified bacterial pneumonia |
| Infection-Pneumonia/Pneumonitis | 10 | J168 | Pneumonia due to other specified infectious organisms |
| Infection-Pneumonia/Pneumonitis | 10 | J180 | Bronchopneumonia unspecified organism |
| Infection-Pneumonia/Pneumonitis | 10 | J181 | Lobar pneumonia unspecified organism |
| Infection-Pneumonia/Pneumonitis | 10 | J189 | Pneumonia unspecified organism |
| Infection-Pneumonia/Pneumonitis | 10 | J690 | Pneumonitis due to inhalation of food and vomit |
| Infection-Pneumonia/Pneumonitis | 10 | J698 | Pneumonitis due to inhalation of other solids and liquids |
| Infection-Pneumonia/Pneumonitis | 10 | J860 | Pyothorax with fistula |
| Infection-Pneumonia/Pneumonitis | 10 | J869 | Pyothorax without fistula |
| Infection-Sepsis | 9 | 38 | Streptococcal septicemia |
| Infection-Sepsis | 9 | 38.1 | Staphylococcal septicemia unspecified |
| Infection-Sepsis | 9 | 38.11 | Methicillin susceptible Staphylococcus aureus septicemia |
| Infection-Sepsis | 9 | 38.12 | Methicillin resistant Staphylococcus aureus septicemia |
| Infection-Sepsis | 9 | 38.19 | Other staphylococcal septicemia |
| Infection-Sepsis | 9 | 38.2 | Pneumococcal septicemia - Streptococcus pneumoniae septicemia- |
| Infection-Sepsis | 9 | 38.4 | Septicemia due to gram-negative organism unspecified |
| Infection-Sepsis | 9 | 38.42 | Septicemia due to escherichia coli - E coli- |
| Infection-Sepsis | 9 | 38.43 | Septicemia due to pseudomonas |
| Infection-Sepsis | 9 | 38.44 | Septicemia due to serratia |
| Infection-Sepsis | 9 | 38.49 | Other septicemia due to gram-negative organisms |
| Infection-Sepsis | 9 | 38.8 | Other specified septicemias |
| Infection-Sepsis | 9 | 38.9 | Unspecified septicemia |
| Infection-Sepsis | 9 | 785.52 | Septic shock |
| Infection-Sepsis | 9 | 790.7 | Bacteremia |
| Infection-Sepsis | 9 | 995.91 | Sepsis |
| Infection-Sepsis | 9 | 995.92 | Severe sepsis |
| Infection-Sepsis | 10 | A403 | Sepsis due to Streptococcus pneumoniae |
| Infection-Sepsis | 10 | A409 | Streptococcal sepsis unspecified |
| Infection-Sepsis | 10 | A4101 | Sepsis due to Methicillin susceptible Staphylococcus aureus |
| Infection-Sepsis | 10 | A4102 | Sepsis due to Methicillin resistant Staphylococcus aureus |
| Infection-Sepsis | 10 | A411 | Sepsis due to other specified staphylococcus |
| Infection-Sepsis | 10 | A412 | Sepsis due to unspecified staphylococcus |
| Infection-Sepsis | 10 | A4150 | Gram-negative sepsis unspecified |
| Infection-Sepsis | 10 | A4151 | Sepsis due to Escherichia coli [E. coli] |
| Infection-Sepsis | 10 | A4152 | Sepsis due to Pseudomonas |
| Infection-Sepsis | 10 | A4153 | Sepsis due to Serratia |
| Infection-Sepsis | 10 | A4159 | Other Gram-negative sepsis |
| Infection-Sepsis | 10 | A4189 | Other specified sepsis |
| Infection-Sepsis | 10 | A419 | Sepsis unspecified organism |
| Infection-Sepsis | 10 | R6520 | Severe sepsis without septic shock |
| Infection-Sepsis | 10 | R6521 | Severe sepsis with septic shock |
| Infection-Sepsis | 10 | R7881 | Bacteremia |
| Infection-Seroma | 9 | 998.51 | Infected postoperative seroma |
| Infection-Urinary Tract | 9 | 599 | Urinary tract infection site not specified |
| Infection-Urinary Tract | 9 | 996.64 | Infection and inflammatory reaction due to indwelling urinary catheter |
| Infection-Urinary Tract | 10 | N390 | Urinary tract infection site not specified |
| Infection-Urinary Tract | 10 | T8351XA | Infection and inflammatory reaction due to indwelling urinary catheter initial encounter |
| Myocardial Infarction | 9 | 410.01 | Acute myocardial infarction of anterolateral wall initial episode of care |
| Myocardial Infarction | 9 | 410.11 | Acute myocardial infarction of other anterior wall initial episode of care |
| Myocardial Infarction | 9 | 410.41 | Acute myocardial infarction of other inferior wall initial episode of care |
| Myocardial Infarction | 9 | 410.71 | Subendocardial infarction initial episode of care |
| Myocardial Infarction | 9 | 410.81 | Acute myocardial infarction of other specified sites initial episode of care |
| Myocardial Infarction | 9 | 410.91 | Acute myocardial infarction of unspecified site initial episode of care |
| Myocardial Infarction | 9 | 411.81 | Acute coronary occlusion without myocardial infarction |
| Myocardial Infarction | 9 | 411.89 | Other acute and subacute forms of ischemic heart disease other |
| Myocardial Infarction | 10 | I2109 | ST elevation (STEMI) myocardial infarction involving other coronary artery of anterior wall |
| Myocardial Infarction | 10 | I2119 | ST elevation (STEMI) myocardial infarction involving other coronary artery of inferior wall |
| Myocardial Infarction | 10 | I2129 | ST elevation (STEMI) myocardial infarction involving other sites |
| Myocardial Infarction | 10 | I213 | ST elevation (STEMI) myocardial infarction of unspecified site |
| Myocardial Infarction | 10 | I214 | Non-ST elevation (NSTEMI) myocardial infarction |
| Myocardial Infarction | 10 | I240 | Acute coronary thrombosis not resulting in myocardial infarction |
| Myocardial Infarction | 10 | I248 | Other forms of acute ischemic heart disease |
| Nausea/Vomiting | 9 | 564.3 | Vomiting following gastrointestinal surgery |
| Nausea/Vomiting | 9 | 787.01 | Nausea with vomiting |
| Nausea/Vomiting | 9 | 787.02 | Nausea alone |
| Nausea/Vomiting | 9 | 787.03 | Vomiting alone |
| Nausea/Vomiting | 10 | K910 | Vomiting following gastrointestinal surgery |
| Nausea/Vomiting | 10 | R110 | Nausea |
| Nausea/Vomiting | 10 | R1110 | Vomiting unspecified |
| Nausea/Vomiting | 10 | R1111 | Vomiting without nausea |
| Nausea/Vomiting | 10 | R1112 | Projectile vomiting |
| Nausea/Vomiting | 10 | R112 | Nausea with vomiting unspecified |
| Reaction | 9 | 995 | Other anaphylactic reaction |
| Reaction | 10 | T782XXA | Anaphylactic shock unspecified initial encounter |
| Reaction | 10 | Y831 | Surgical operation with implant of artificial internal device as the cause of abnormal reaction of the patient or of later complication |
| Reaction | 10 | Y838 | Other surgical procedures as the cause of abnormal reaction of the patient or of later complication without mention of misadventure |
| Reaction | 10 | Y839 | Surgical procedure unspecified as the cause of abnormal reaction of the patient or of later complication without mention of misadventure |
| Reaction | 10 | Y846 | Urinary catheterization as the cause of abnormal reaction of the patient or of later complication without mention of misadventure at the time |
| Reaction | 10 | Y849 | Medical procedure unspecified as the cause of abnormal reaction of the patient or of later complication without mention of misadventure |
| Respiratory Failure | 9 | 518 | Pulmonary collapse |
| Respiratory Failure | 9 | 518.51 | Acute respiratory failure following trauma and surgery |
| Respiratory Failure | 9 | 518.52 | Other pulmonary insufficiency not elsewhere classified following trauma and surgery |
| Respiratory Failure | 9 | 518.53 | Acute and chronic respiratory failure following trauma and surgery |
| Respiratory Failure | 9 | 518.81 | Acute respiratory failure |
| Respiratory Failure | 9 | 518.83 | Chronic respiratory failure |
| Respiratory Failure | 9 | 518.84 | Acute and chronic respiratory failure |
| Respiratory Failure | 10 | J951 | Acute pulmonary insufficiency following thoracic surgery |
| Respiratory Failure | 10 | J952 | Acute pulmonary insufficiency following nonthoracic surgery |
| Respiratory Failure | 10 | J953 | Chronic pulmonary insufficiency following surgery |
| Respiratory Failure | 10 | J95821 | Acute postprocedural respiratory failure |
| Respiratory Failure | 10 | J95822 | Acute and chronic postprocedural respiratory failure |
| Respiratory Failure | 10 | J9600 | Acute respiratory failure unspecified whether with hypoxia or hypercapnia |
| Respiratory Failure | 10 | J9610 | Chronic respiratory failure unspecified whether with hypoxia or hypercapnia |
| Respiratory Failure | 10 | J9620 | Acute and chronic respiratory failure unspecified whether with hypoxia or hypercapnia |
| Respiratory Failure | 10 | J9690 | Respiratory failure unspecified unspecified whether with hypoxia or hypercapnia |
| Respiratory Failure | 10 | J9811 | Atelectasis |
| Respiratory Failure | 10 | J9819 | Other pulmonary collapse |
| Retained Foreign Body | 9 | 998.4 | Foreign body accidentally left during a procedure |
| Retained Foreign Body | 10 | T81509A | Unspecified complication of foreign body accidentally left in body following unspecified procedure initial encounter |
| Retained Foreign Body | 10 | T81519A | Adhesions due to foreign body accidentally left in body following unspecified procedure initial encounter |
| Retained Foreign Body | 10 | T81529A | Obstruction due to foreign body accidentally left in body following unspecified procedure initial encounter |
| Retained Foreign Body | 10 | T81539A | Perforation due to foreign body accidentally left in body following unspecified procedure initial encounter |
| Seroma | 9 | 998.13 | Seroma complicating a procedure |
| Seroma | 10 | T888XXA | Other specified complications of surgical and medical care not elsewhere classified initial encounter |
| Stroke | 9 | 430 | Subarachnoid hemorrhage |
| Stroke | 9 | 431 | Intracerebral hemorrhage |
| Stroke | 9 | 432.9 | Unspecified intracranial hemorrhage |
| Stroke | 9 | 433.11 | Occlusion and stenosis of carotid artery with cerebral infarction |
| Stroke | 9 | 433.81 | Occlusion and stenosis of other specified precerebral artery with cerebral infarction |
| Stroke | 9 | 434.01 | Cerebral thrombosis with cerebral infarction |
| Stroke | 9 | 434.11 | Cerebral embolism with cerebral infarction |
| Stroke | 9 | 434.91 | Cerebral artery occlusion unspecified with cerebral infarction |
| Stroke | 9 | 997.02 | Iatrogenic cerebrovascular infarction or hemorrhage |
| Stroke | 10 | I609 | Nontraumatic subarachnoid hemorrhage unspecified |
| Stroke | 10 | I619 | Nontraumatic intracerebral hemorrhage unspecified |
| Stroke | 10 | I629 | Nontraumatic intracranial hemorrhage unspecified |
| Stroke | 10 | I63139 | Cerebral infarction due to embolism of unspecified carotid artery |
| Stroke | 10 | I63239 | Cerebral infarction due to unspecified occlusion or stenosis of unspecified carotid arteries |
| Stroke | 10 | I6330 | Cerebral infarction due to thrombosis of unspecified cerebral artery |
| Stroke | 10 | I6340 | Cerebral infarction due to embolism of unspecified cerebral artery |
| Stroke | 10 | I6350 | Cerebral infarction due to unspecified occlusion or stenosis of unspecified cerebral artery |
| Stroke | 10 | I6359 | Cerebral infarction due to unspecified occlusion or stenosis of other cerebral artery |
| Stroke | 10 | I97811 | Intraoperative cerebrovascular infarction during other surgery |
| Stroke | 10 | I97821 | Postprocedural cerebrovascular infarction during other surgery |
| Subcutaneous Emphysema | 9 | 998.81 | Emphysema - subcutaneous- - surgical- resulting from procedure |
| Subcutaneous Emphysema | 10 | T8182XA | Emphysema (subcutaneous) resulting from a procedure initial encounter |
| Surgical Injury | 9 | 998.2 | Accidental puncture or laceration during a procedure not elsewhere classified |
| Surgical Injury | 10 | G9748 | Accidental puncture and laceration of other nervous system organ or structure during a nervous system procedure |
| Surgical Injury | 10 | G9749 | Accidental puncture and laceration of other nervous system organ or structure during other procedure |
| Surgical Injury | 10 | I9751 | Accidental puncture and laceration of a circulatory system organ or structure during a circulatory system procedure |
| Surgical Injury | 10 | I9752 | Accidental puncture and laceration of a circulatory system organ or structure during other procedure |
| Surgical Injury | 10 | M96820 | Accidental puncture and laceration of a musculoskeletal structure during a musculoskeletal system procedure |
| Surgical Injury | 10 | M96821 | Accidental puncture and laceration of a musculoskeletal structure during other procedure |
| Thrombophlebitis | 9 | 451.19 | Phlebitis and thrombophlebitis of deep veins of lower extremities other |
| Thrombophlebitis | 9 | 451.2 | Phlebitis and thrombophlebitis of lower extremities unspecified |
| Thrombophlebitis | 10 | I80209 | Phlebitis and thrombophlebitis of unspecified deep vessels of unspecified lower extremity |
| Thrombophlebitis | 10 | I803 | Phlebitis and thrombophlebitis of lower extremities unspecified |
| Thrombophlebitis-DVT | 9 | 453.4 | Acute venous embolism and thrombosis of unspecified deep vessels of lower extremity |
| Thrombophlebitis-DVT | 9 | 453.41 | Acute venous embolism and thrombosis of deep vessels of proximal lower extremity |
| Thrombophlebitis-DVT | 9 | 453.42 | Acute venous embolism and thrombosis of deep vessels of distal lower extremity |
| Thrombophlebitis-DVT | 10 | I82409 | Acute embolism and thrombosis of unspecified deep veins of unspecified lower extremity |
| Thrombophlebitis-DVT | 10 | I82419 | Acute embolism and thrombosis of unspecified femoral vein |
| Thrombophlebitis-DVT | 10 | I82429 | Acute embolism and thrombosis of unspecified iliac vein |
| Thrombophlebitis-DVT | 10 | I82439 | Acute embolism and thrombosis of unspecified popliteal vein |
| Thrombophlebitis-DVT | 10 | I82449 | Acute embolism and thrombosis of unspecified tibial vein |
| Thrombophlebitis-DVT | 10 | I82499 | Acute embolism and thrombosis of other specified deep vein of unspecified lower extremity |
| Thrombophlebitis-DVT | 10 | I824Y9 | Acute embolism and thrombosis of unspecified deep veins of unspecified proximal lower extremity |
| Thrombophlebitis-DVT | 10 | I824Z9 | Acute embolism and thrombosis of unspecified deep veins of unspecified distal lower extremity |
| TIA | 9 | 435.9 | Unspecified transient cerebral ischemia |
| TIA | 10 | G459 | Transient cerebral ischemic attack unspecified |
| TIA | 10 | I67848 | Other cerebrovascular vasospasm and vasoconstriction |
| Transfusion Risk | 9 | 999.8 | Transfusion reaction unspecified |
| Transfusion Risk | 9 | 999.85 | Delayed hemolytic transfusion reaction incompatibility unspecified |
| Transfusion Risk | 9 | 999.89 | Other transfusion reaction |
| Transfusion Risk | 10 | T8089XA | Other complications following infusion transfusion and therapeutic injection initial encounter |
| Transfusion Risk | 10 | T80911A | Delayed hemolytic transfusion reaction unspecified incompatibility initial encounter |
| Transfusion Risk | 10 | T8092XA | Unspecified transfusion reaction initial encounter |
